# Supplementary material for: Optimizing tuberculosis treatment efficacy: Comparing the standard regimen with Moxifloxacin-containing regimens
Source: PLoS Comput Biol. 2023 Jun 15;19(6):e1010823. doi: 10.1371/journal.pcbi.1010823 (PMC10306236; doi:10.1371/journal.pcbi.1010823)
Supplement: S1 Appendix — Fig A. Visualization of simulated NHP granuloma FDG avidity using GranSim. The structure indicates FDG avidity of a simulated granuloma using GranSim. The brightness scale of each microcompartment on the grid is calculated based on inflammation within GranSim measured here by TNF concentration levels and a weighted sum of each proinflammatory immune cell in the granuloma. See Methods for details. Fig B. Schematic of Pareto Front Optimization. The non-dominating solutions, i.e., the Pareto set (x’s) for a problem with two objectives f1(x) and f2(x). The solid red line represents the Pareto front. Any solution that lies in the red shaded area would be a part of the Pareto set, whereas solutions in the blue shaded area would dominate the preexisting Pareto set and, hence, replace it [1]. Fig C. Comparing 3-way simulated combinations of HRZEM using GranSim. Comparison of simulations for (A-C) fraction of unsterilized granulomas and (D-F) sterilization times of 3-way combinations of HRZEM for (A and D) 100 high-CFU, (B and E) 100 low-CFU and (C and F) a combination of 100 high- and 100 low-CFU granulomas. (A-C) Moxifloxacin-containing regimens sterilize granulomas a lot faster initially than regimens without moxifloxacin in all cases, and this difference is more pronounced for (B) low-CFU granulomas. (D-F) Sterilization times shows a similar trend: granulomas treated with regimens containing moxifloxacin (red boxes) are cleared in a shorter time frames on average than regimens not containing moxifloxacin (black boxes). We performed significance tests between each possible pair of regimens with (red boxes) and without (black boxes) including moxifloxacin and show that regimens containing moxifloxacin are significantly more efficacious than regimens that do not include moxifloxacin (*p<0.0001, one-tailed paired t-test). The central red lines in box plots represent the median, whereas the bottom and the top edges of boxes represent 25th and 75th percentiles, respectively. F [file pcbi.1010823.s001.docx]

**S1 Appendix**

Optimizing efficacy of treatments for tuberculosis: comparing the standard regimen with Moxifloxacin-containing regimens

Maral Budak, Joseph M. Cicchese, Pauline Maiello, H. Jacob Borish, Alexander G. White, Harris B. Chishti, Jaime Tomko, L. James Frye, Daniel Fillmore, Kara Kracinovsky, Jennifer Sakal, Charles A. Scanga, Philana Ling Lin, Véronique Dartois, Jennifer J. Linderman, JoAnne L. Flynn, Denise E. Kirschner

Below are additional figures and tables referenced in the main text of the paper. All data generated or analyzed during this study are included in this published article and are available upon request.


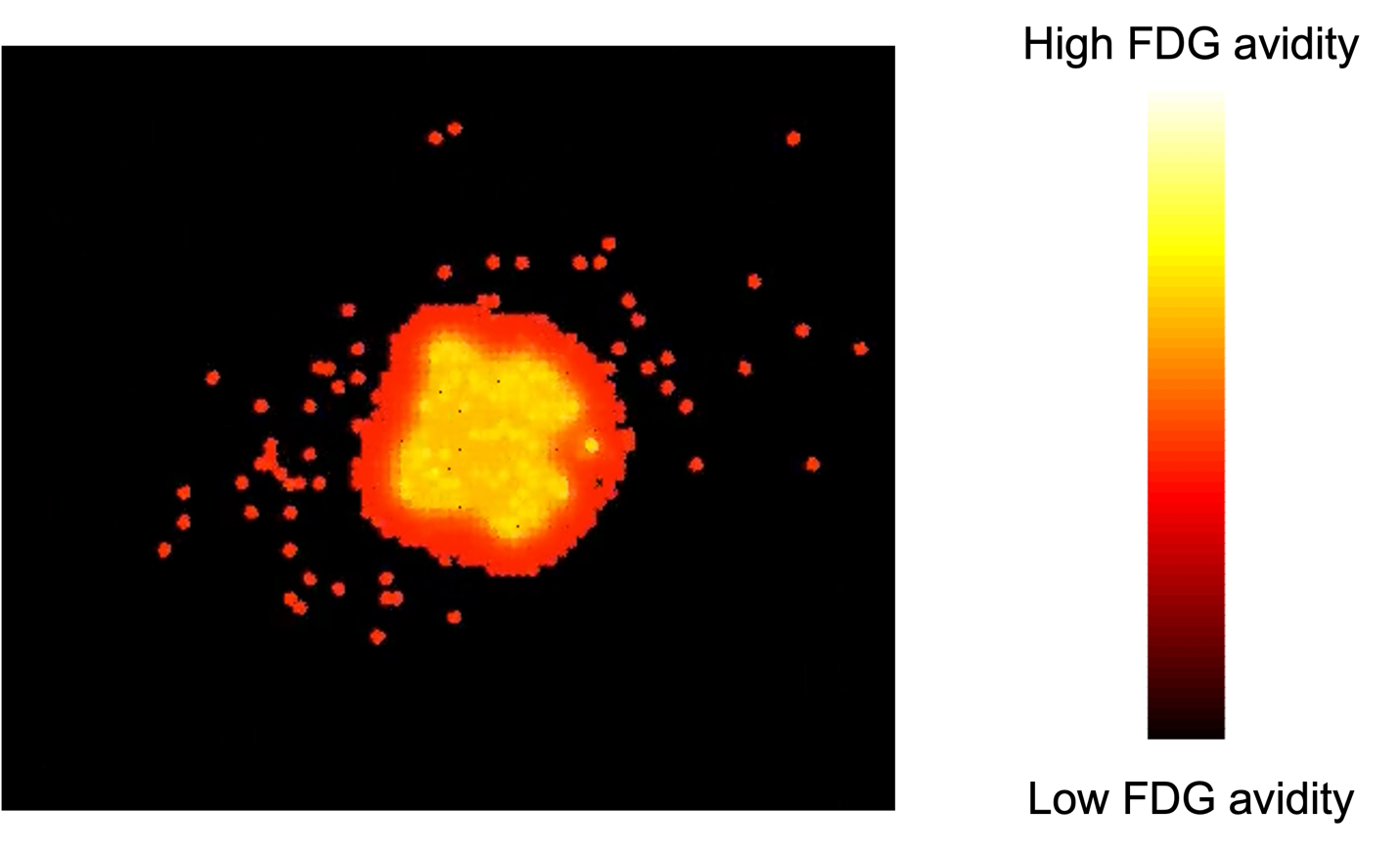


**Fig A.** **Visualization of simulated NHP granuloma FDG avidity using *GranSim*.** The structure indicates *FDG avidity* of a simulated granuloma using *GranSim*. The brightness scale of each microcompartment on the grid is calculated based on inflammation within *GranSim* measured here by TNF concentration levels and a weighted sum of each proinflammatory immune cell in the granuloma. See Methods for details.

**Pareto optimization**

We can illustrate the Pareto set (x’s in Fig B) by plotting one of the objective functions (f_2_(x) in Fig B) as a function of the other objective (f_1_(x) in Fig B). By this way, we can determine the Pareto front (solid red line in Fig B), the series of horizontal and vertical lines that joins the Pareto set. Any data point that lies in the red shaded area in Fig B would be unable to dominate the current Pareto set and, hence, would be a part of the Pareto set. These are the optimal solutions to the problem.


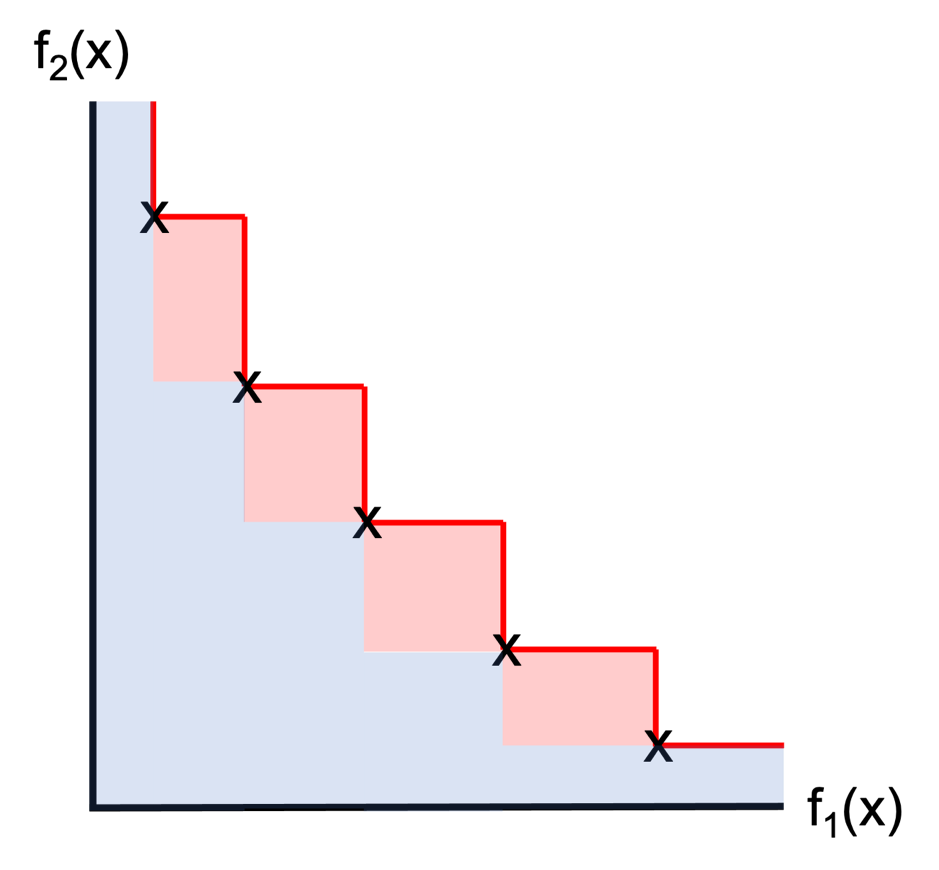


**Fig B.** **Schematic of Pareto Front Optimization.** The non-dominating solutions, i.e., the Pareto set (x’s) for a problem with two objectives f_1_(x) and f_2_(x). The solid red line represents the Pareto front. Any solution that lies in the red shaded area would be a part of the Pareto set, whereas solutions in the blue shaded area would dominate the preexisting Pareto set and, hence, replace it [1].


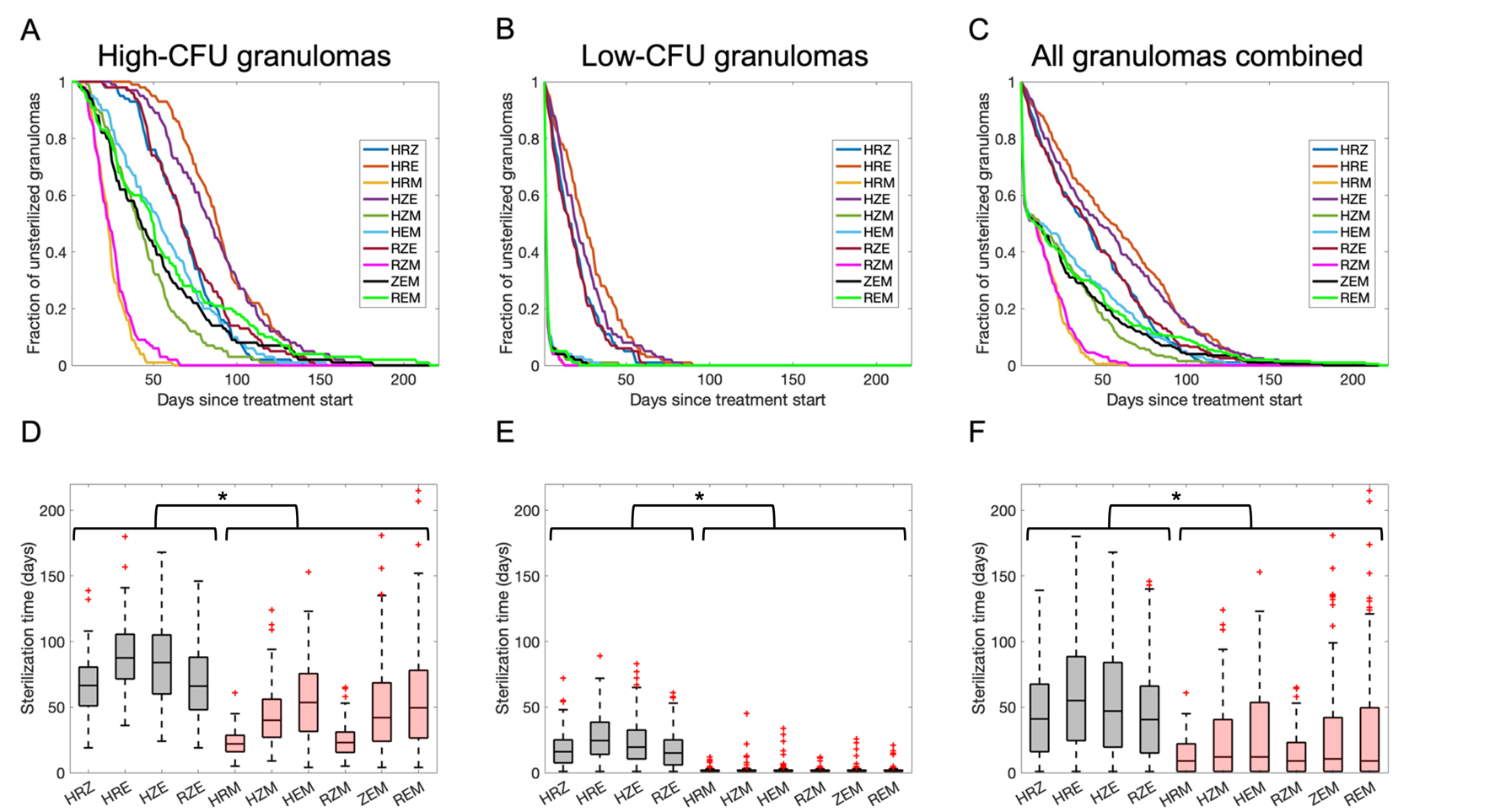


**Fig C.** **Comparing** **3-way simulated combinations of HRZEM using *GranSim.*** Comparison of simulations for (A-C) fraction of unsterilized granulomas and (D-F) sterilization times of 3-way combinations of HRZEM for (A and D) 100 high-CFU, (B and E) 100 low-CFU and (C and F) a combination of 100 high- and 100 low-CFU granulomas. (A-C) Moxifloxacin-containing regimens sterilize granulomas a lot faster initially than regimens without moxifloxacin in all cases, and this difference is more pronounced for (B) low-CFU granulomas. (D-F) Sterilization times shows a similar trend:  granulomas treated with regimens containing moxifloxacin (red boxes) are cleared in a shorter time frames on average than regimens not containing moxifloxacin (black boxes). We performed significance tests between each possible pair of regimens with (red boxes) and without (black boxes) including moxifloxacin and show that regimens containing moxifloxacin are significantly more efficacious than regimens that do not include moxifloxacin (*p<0.0001, one-tailed paired t-test). The central red lines in box plots represent the median, whereas the bottom and the top edges of boxes represent 25^th^ and 75^th^ percentiles, respectively.

**
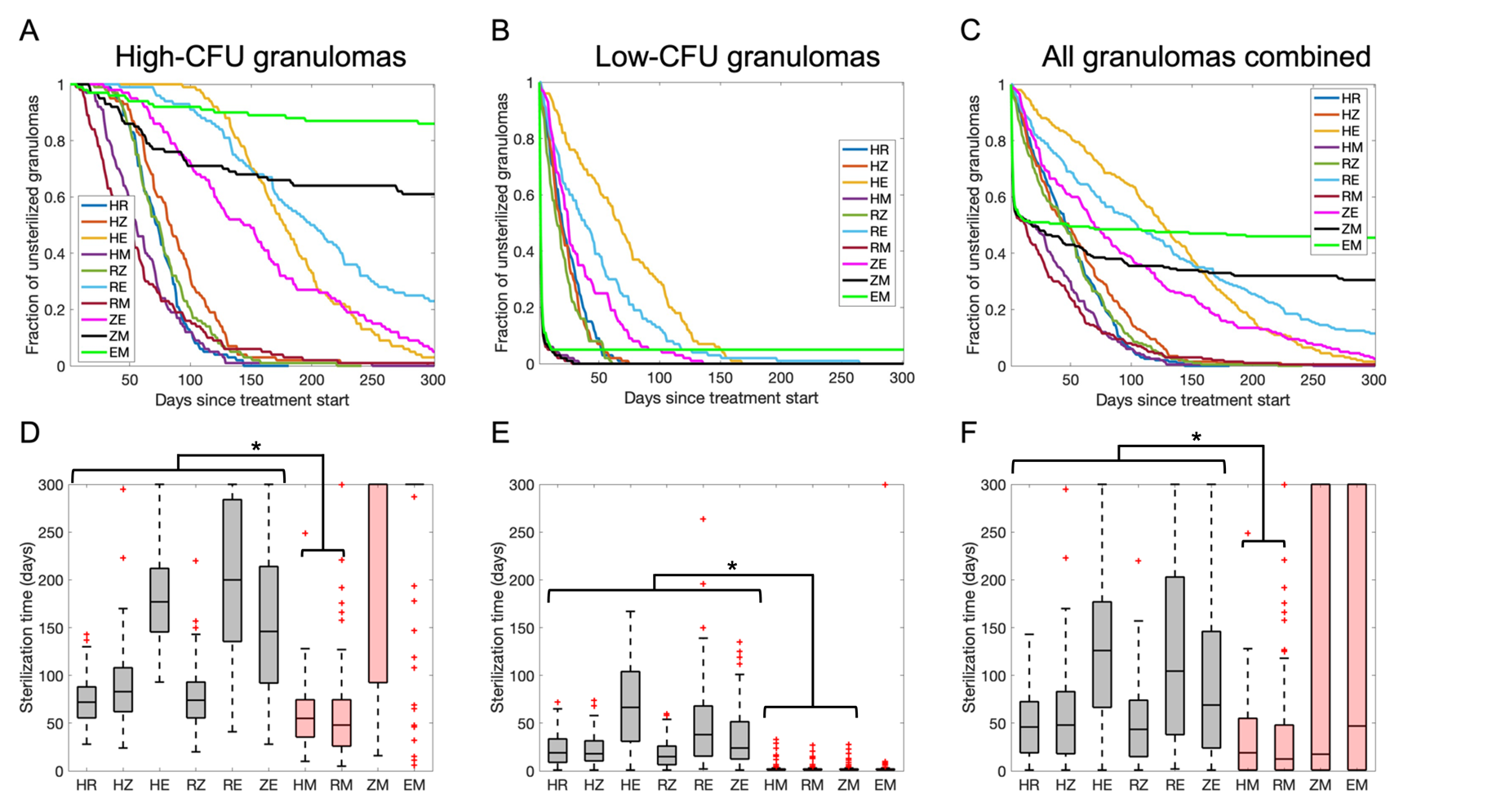
**

**Fig D.** **Comparing** **2-way simulated combinations of HRZEM using *GranSim.*** Comparison of simulations for (A-C) fraction of unsterilized granulomas and (D-F) sterilization times of 2-way combinations of HRZEM for (A and D) 100 high-CFU, (B and E) 100 low-CFU and (C and F) a combination of 100 high- and 100 low-CFU granulomas. (A-C) Moxifloxacin-containing regimens sterilize (B) low-CFU granulomas faster initially than regimens not containing moxifloxacin in all cases, but the same trend does not always hold for (A) high-CFU granulomas and (C) the combination of high- and low-CFU granulomas. (D-F) Sterilization times averaged over all granulomas for (D) high-CFU and (E) low-CFU granulomas, and (F) both groups combined (black boxes: regimens with no moxifloxacin, red boxes: regimens with moxifloxacin). We performed significance tests between each possible pair of regimens with (red boxes) and without (black boxes) including moxifloxacin and showed that HM and RM are significantly more efficacious than regimens not including moxifloxacin (*p<0.005, one-tailed paired t-test). The central red lines in box plots represent the median, whereas the bottom and the top edges of boxes represent 25^th^ and 75^th^ percentiles, respectively.


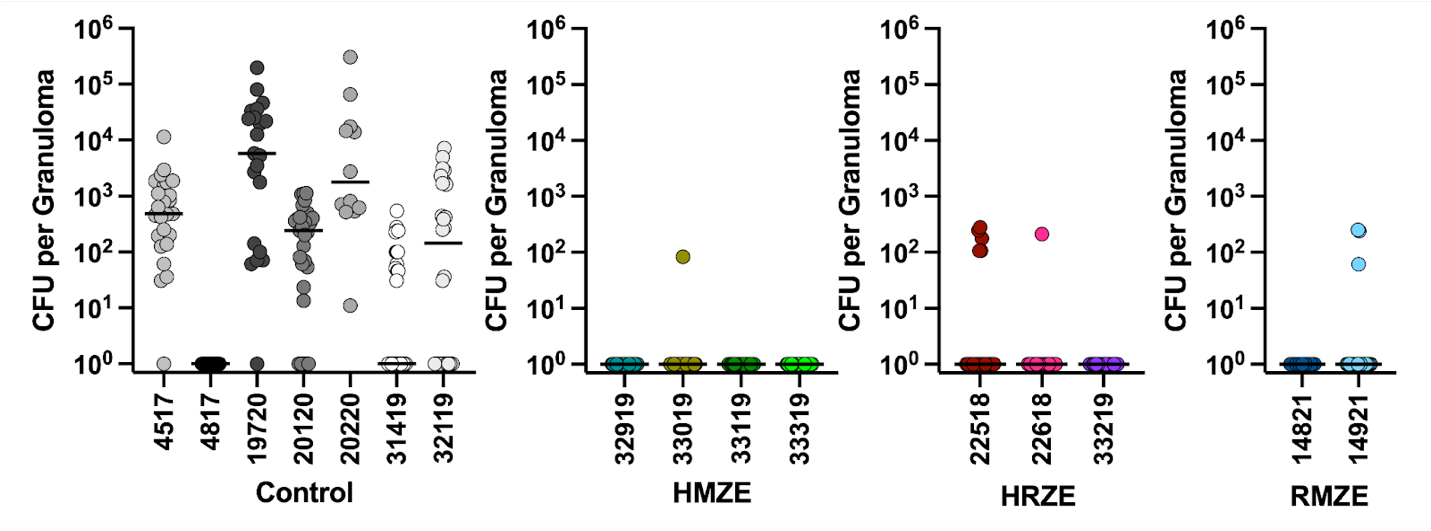


**Fig E.** **Data derived from NHP studies on total lung CFU**. The total CFU per NHP granulomas after NHP are treated with the corresponding regimens for two months demonstrate that most granulomas are sterilized by all regimens compared to the control (n=107 granulomas from 7 animals in the control group, n=117 granulomas from 4 animals in HMZE group, n=52 granulomas from 3 animals in HRZE group, n=34 granulomas from 2 animals in RMZE group).


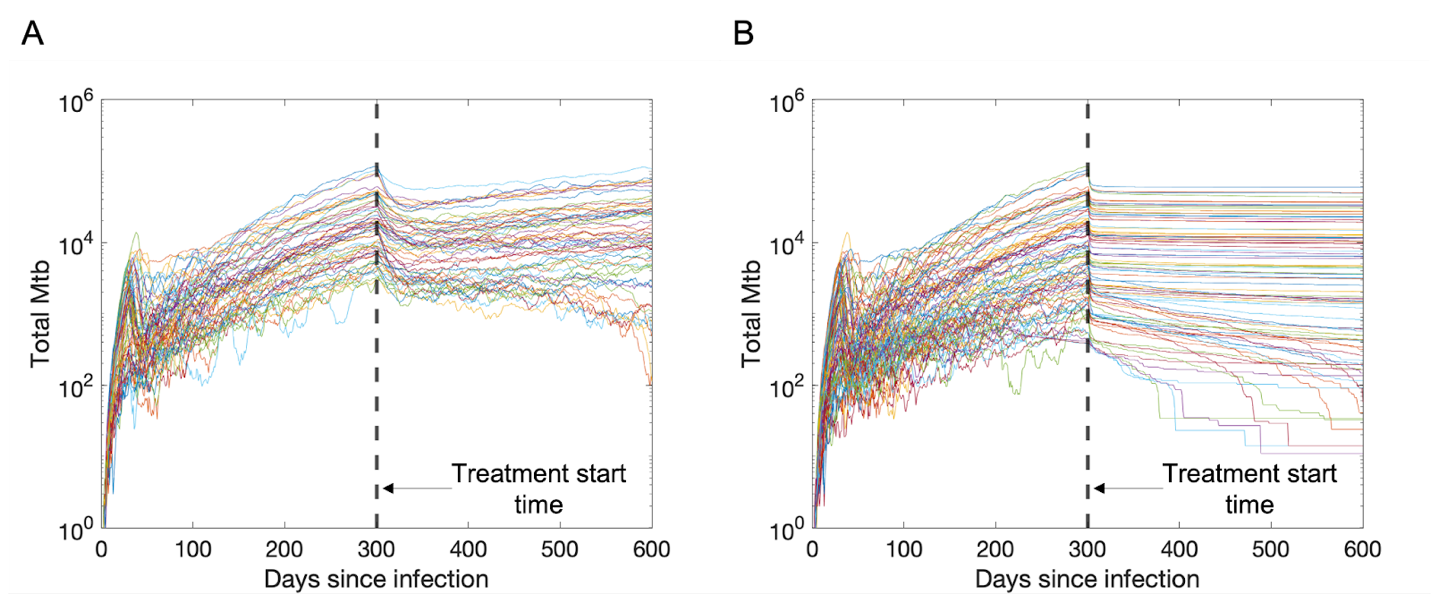

**Fig F.** **Simulation showing possibility of** **Paucibacilliary TB for 2 regimen treatments.** Total number of Mtb of high-CFU granulomas when treated with (A) ZM and (B) EM for 300 days. Treatment starts 300 days after the infection. Each line represents a granuloma simulation in *GranSim*.

**Table A.** **Fractional inhibitory concentration (FIC) values of each regimen as predicted by INDIGO-MTB [2].** These values are used in the drug combinations.

|  | **Regimen** | **FIC** |
| --- | --- | --- |
| **4-way** | HRZE | 0.82 |
|  | HMZE | 1.08 |
|  | RMZE | 1.07 |
|  | HRMZ | 0.97 |
|  | HRME | 1.06 |
| **3-way** | HRZ | 0.81 |
|  | HRE | 0.87 |
|  | HZE | 0.90 |
|  | RZE | 0.85 |
|  | HRM | 1.06 |
|  | HMZ | 1.15 |
|  | HME | 1.10 |
|  | RMZ | 1.06 |
|  | REM | 1.08 |
|  | ZEM | 1.05 |
| **2-way** | HR | 1.37 |
|  | HZ | 1.11 |
|  | HE | 1.13 |
|  | RZ | 0.91 |
|  | ZE | 0.99 |
|  | RE | 0.86 |
|  | HM | 2.01 |
|  | RM | 2.27 |
|  | ZM | 1.93 |
|  | EM | 2.06 |

**Table B.** **Predicted** **dosages for each antibiotic in the HRZM regimen that are optimal for minimizing dose and sterilization time (red dots in Fig 7A).** Red row indicates the regimen with CDC-recommended doses for each antibiotic (green dot in Fig 7A) [3]. The row labeled with a triangle indicates an optimal 3-way combination, where the optimal dose of Z is predicted as 0.

| **Avg Sterilization Time (days)** | **Total dose (mg/kg)** | **H** | **R** | **Z** | **M** |
| --- | --- | --- | --- | --- | --- |
| 12.0 | 47 | 5 | 10 | 25 | 7 |
| 6.2 | 79.2 | 7.2 | 19.5 | 43.6 | 9 |
| 6.2 | 63.3 | 9.9 | 19.7 | 19.7 | 14 |
| 6.7 | 37 | 8.8 | 19.7 | 0.6 | 7.9 |
| 8.6 | 36.2 | 1.3 | 15.9 | 9 | 10.1 |
| 8.7 | 32.3 | 8.7 | 7.7 | 5.1 | 10.8 |
| 9.2 | 30.1 | 10 | 9.7 | 3.9 | 6.5 |
| 20.5 | 16.4 | 9.1 | 0.9 | 1.2 | 5.2 |
| 36.8 | 12 | 3.3 | 4.1 | 1.3 | 3.2 |
| 54.6 | 9.9 | 3.1 | 4.6 | 0.3 | 1.9 |
| 116.0 | 6.8 | 0.9 | 5.1 | 0 | 0.8 |

**Table C.** **Predicted dosages of each antibiotic in HRME regimen that is optimal for dose and sterilization times (Red dots Fig 7B).** Red row indicates the regimen with CDC-recommended doses for each antibiotic (green dot in Fig 7B) [3]. The rows labeled with a triangle indicate an optimal 3-way combination, where the optimal dose of E is predicted as 0.

| **Avg Sterilization Time (days)** | **Total dose (mg/kg)** | **H** | **R** | **M** | **E** |
| --- | --- | --- | --- | --- | --- |
| 14.0 | 42 | 5 | 10 | 7 | 20 |
| 4.6 | 61.8 | 9.7 | 19 | 13.6 | 19.5 |
| 5.1 | 61.1 | 10 | 15.7 | 12.3 | 23.1 |
| 5.1 | 55.2 | 6.1 | 20 | 12.3 | 16.8 |
| 5.5 | 54.1 | 7.5 | 13.9 | 13 | 19.7 |
| 6.1 | 40.8 | 9 | 11.3 | 13.3 | 7.2 |
| 7.5 | 29.6 | 5.5 | 10.1 | 14 | 0 |
| 9.5 | 23.4 | 6.7 | 9.2 | 7.5 | 0 |
| 29.7 | 16.8 | 5.4 | 5.1 | 3.2 | 3.1 |
| 106.7 | 7.2 | 0.1 | 0.8 | 2.7 | 3.6 |

**Table D.** **Predicted dosages of each antibiotic in HMZE regimen that are optimal for dose and sterilization times (Red dots Fig 7C).** Red row indicates the regimen with CDC-recommended doses for each antibiotic (green dot in Fig 7C) [3]. The row labeled with a triangle indicates an optimal 3-way combination, where the optimal dose of E is predicted as 0.

| **Avg Sterilization Time (days)** | **Total dose (mg/kg)** | **H** | **M** | **Z** | **E** |
| --- | --- | --- | --- | --- | --- |
| 18.7 | 57 | 5 | 7 | 25 | 20 |
| 6.7 | 91.2 | 9.7 | 13.3 | 48.7 | 19.5 |
| 7.6 | 90.0 | 7.2 | 13.6 | 43.6 | 25.6 |
| 8.1 | 85.8 | 7.7 | 11.0 | 49.6 | 17.5 |
| 9.1 | 75.1 | 7.5 | 11.4 | 38.9 | 17.3 |
| 9.3 | 52.2 | 8.7 | 11.8 | 31.2 | 0.5 |
| 10.9 | 51.3 | 9.8 | 9.9 | 18.7 | 12.9 |
| 13.8 | 21.1 | 8.2 | 9.3 | 2.6 | 1.0 |
| 45.6 | 14.6 | 1.8 | 11.9 | 0.9 | 0 |
| 63.2 | 13.5 | 1.1 | 9.9 | 1.7 | 0.8 |
| 114.3 | 5.2 | 2.5 | 1.3 | 0.3 | 1.1 |
| 140.5 | 4.1 | 1.7 | 1.2 | 0.6 | 0.6 |

**Table E.** **Predicted dosages of each antibiotic in RMZE regimen that are optimal for dose and sterilization times (Red dots Fig 7D).** Red row indicates the regimen with CDC-recommended doses for each antibiotic (green dot in Fig 7D) [3]. The rows labeled with a triangle indicate an optimal 3-way combination, where the optimal dose of E or Z is predicted as 0.

| **Avg Sterilization Time (days)** | **Total dose (mg/kg)** | **R** | **M** | **Z** | **E** |
| --- | --- | --- | --- | --- | --- |
| 17.0 | 62 | 10 | 7 | 25 | 20 |
| 5.1 | 121.9 | 13.9 | 20.0 | 50.0 | 38.0 |
| 5.1 | 110.6 | 13.2 | 18.8 | 46.3 | 32.3 |
| 5.2 | 100.8 | 13.6 | 19.0 | 48.7 | 19.5 |
| 5.5 | 95.4 | 13.3 | 19.8 | 27.1 | 35.2 |
| 5.8 | 79.9 | 13.1 | 18.8 | 40.2 | 7.8 |
| 6.6 | 75.9 | 14.0 | 14.6 | 28.6 | 18.7 |
| 7.4 | 61.8 | 10.4 | 20.0 | 25.8 | 5.6 |
| 8.8 | 47.2 | 8.7 | 18.4 | 16 | 4.1 |
| 10.6 | 45.4 | 9.3 | 9.2 | 26.9 | 0 |
| 11.0 | 28.4 | 11.5 | 13.3 | 2.6 | 1.0 |
| 17.3 | 23.0 | 7.8 | 12.7 | 0 | 2.5 |
| 53.7 | 13.7 | 4.7 | 6.9 | 2.1 | 0 |
| 97.5 | 7.3 | 3.0 | 1.9 | 2.3 | 0.1 |

**Table F.** **Predicted dosages of each antibiotic in HRZE regimen that are optimal for dose and sterilization times (Red dots Fig 7E).** Red row indicates the regimen with CDC-recommended doses for each antibiotic (green dot in Fig 7E) [3].

| **Avg Sterilization Time (days)** | **Total dose (mg/kg)** | **H** | **R** | **Z** | **E** |
| --- | --- | --- | --- | --- | --- |
| 33.6 | 60 | 5 | 10 | 25 | 20 |
| 16.3 | 96.9 | 9.7 | 19.0 | 48.7 | 19.5 |
| 18.0 | 95.9 | 7.2 | 19.5 | 43.6 | 25.6 |
| 20.0 | 90.5 | 5.1 | 20.0 | 44.9 | 20.5 |
| 21.0 | 78.2 | 4.4 | 15.7 | 49.9 | 8.2 |
| 21.4 | 74.9 | 9.0 | 11.3 | 47.4 | 7.2 |
| 25.8 | 74.1 | 4.4 | 17.4 | 25.6 | 26.7 |
| 27.3 | 38.7 | 4.7 | 19.0 | 14.2 | 0.8 |
| 37.3 | 34.9 | 5.9 | 10.8 | 14.1 | 4.1 |
| 38.6 | 25.1 | 8.2 | 13.3 | 2.6 | 1.0 |
| 40.3 | 20.0 | 3.8 | 13.8 | 2.3 | 0.1 |
| 90.3 | 10.6 | 5.5 | 2.5 | 1.8 | 0.8 |
| 169.3 | 4.0 | 0.2 | 1.8 | 0.5 | 1.5 |

**References**

1. Keane AJ. Statistical improvement criteria for use in multiobjective design optimization. AIAA Journal; 2006. p. 879-91.

2. Ma S, Jaipalli S, Larkins-Ford J, Lohmiller J, Aldridge BB, Sherman DR, et al. Transcriptomic Signatures Predict Regulators of Drug Synergy and Clinical Regimen Efficacy against Tuberculosis. mBio. 2019;10(6). Epub 20191112. doi: 10.1128/mBio.02627-19. PubMed PMID: 31719182; PubMed Central PMCID: PMCPMC6851285.

3. Nahid P, Dorman SE, Alipanah N, Barry PM, Brozek JL, Cattamanchi A, et al. Executive Summary: Official American Thoracic Society/Centers for Disease Control and Prevention/Infectious Diseases Society of America Clinical Practice Guidelines: Treatment of Drug-Susceptible Tuberculosis. Clin Infect Dis. 2016;63(7):853-67. doi: 10.1093/cid/ciw566. PubMed PMID: 27621353; PubMed Central PMCID: PMCPMC6366011.
