# Supplementary material for: Optimizing tuberculosis treatment efficacy: Comparing the standard regimen with Moxifloxacin-containing regimens
Source: PLoS Comput Biol. 2023 Jun 15;19(6):e1010823. doi: 10.1371/journal.pcbi.1010823 (PMC10306236; doi:10.1371/journal.pcbi.1010823)
Supplement: S1 Text — Fig A. GranSim Calibrated to non-human primate data from Flynn lab [5,6]. (A) T cell and (B) macrophage counts from a new set of granulomas sampled from calibrated parameter ranges span the range of in vivo data. Black dots are in vivo data from NHP granulomas, blue lines are the maximum, mean and minimum (from top to bottom) values of corresponding simulated cell counts from GranSim simulations, and the blue shaded area is between the minimum and maximum. Fig B. Schematic representation of how antibiotics are partitioned within a grid microcompartment within GranSim. Within a microcompartment, antibiotics can be caseum-bound (DCcaseum), be located within macrophages (DCmac) or be free without binding to anything (DCfree), depending on the availability of caseous tissue and macrophages within that microgrid. (DCcaseum: drug concentration bound to caseum, DCmac: drug concentration within a macrophage, DCfree: free drug concentration, green circle: macrophage, brown bleb: caseum, black circles: replicating extracellular Mtb, light green circle: intracellular Mtb, tan circle: nonreplicating Mtb). Fig C. Calibration of GranSim PK/PD to Moxifloxacin datasets. Calibration of moxifloxacin (MXF) plasma and tissue PK to temporal (black dots in A-D) and spatial (see Fig 2B in [8]) data from human granulomas. Black lines in A-D are the maximum, mean and minimum (from top to bottom) values of MXF concentrations resulting from 100 GranSim simulations, and the black shaded area is between the minimum and maximum. Average simulated MXF concentrations in (A) blood, (B) granuloma, (C) uninvolved lung and (D) caseum agree with human data. (E) Spatial analysis of how MXF is distributed within a granuloma in GranSim indicates that MXF does not easily diffuse into caseum, which is consistent with MALDI-MS imaging of granulomas in [8]. All other drugs were calibrated using this same approach. Table A. Pharmacodynamic (PD) parameters for each drug and Mtb type and sources for bactericidal [file pcbi.1010823.s002.docx]

**S1 Text.** **Changes to *GranSim* and PK/PD modeling that are included in this next-generation version.**

*GranSim* is continuously updated based on biology. Since the last published version[1], we have recalibrated *GranSim* and PK/PD parameters of all included drugs as well as the PD model, as follows.

1.
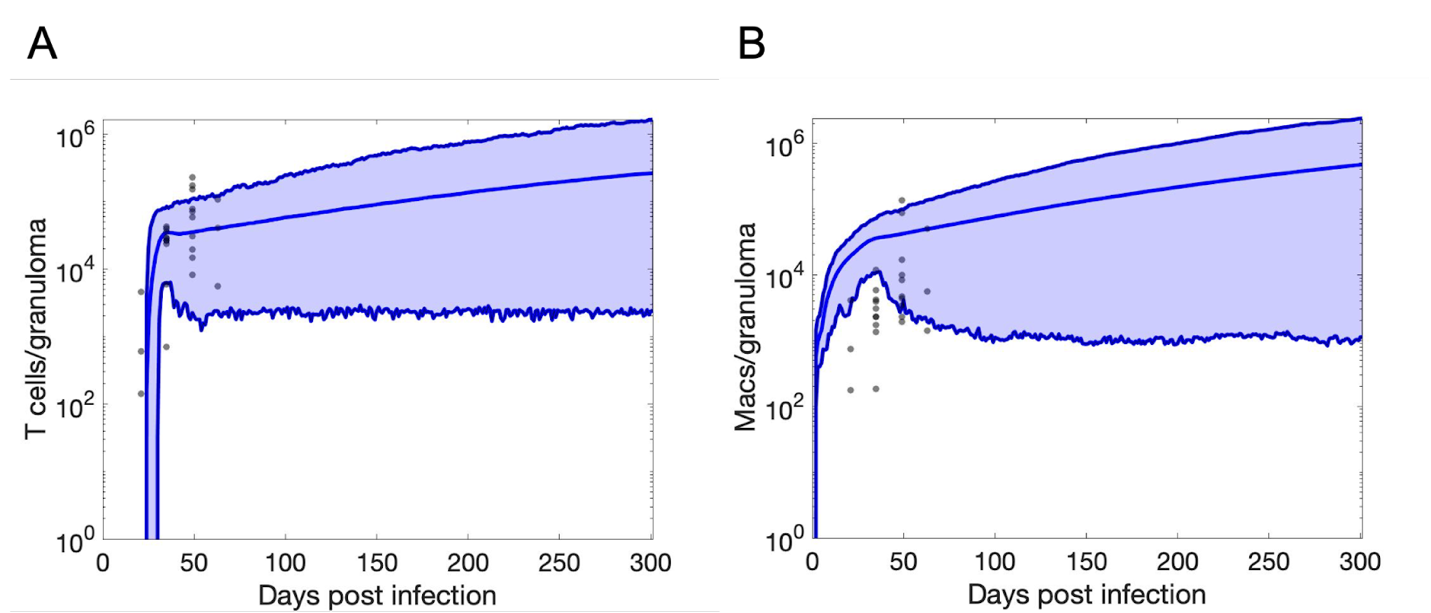
We recalibrate *GranSim* to the most recent CFU (Fig 1) and immune cell counts, i.e., T cells (panel A in Fig A) and macrophages (panel B Fig A), from NHP granulomas [2, 3].

**Fig A.** ***GranSim*** **Calibrated to non-human primate data from Flynn lab [2, 3].** (A) T cell and (B) macrophage counts from a new set of granulomas sampled from calibrated parameter ranges span the range of *in vivo* data. Black dots are *in vivo* data from NHP granulomas, blue lines are the maximum, mean and minimum (from top to bottom) values of corresponding simulated cell counts from *GranSim* simulations, and the blue shaded area is between the minimum and maximum.

1. At every grid microcompartment, we partition antibiotics into different subcompartments (Fig B). They can be bound to caseum (*DC_caseum_*), taken up by macrophages (*DC_mac_*) or stay unbound on the microcompartment (*DC_free_*). We use the *in vitro* measures ‘caseum unbound fraction’ [4] and ‘macrophage accumulation ratio’ [5] to calibrate the ratios *DC_mac_/DC_free_* and *DC_caseum_/DC_free_* that are used in the process of partitioning.


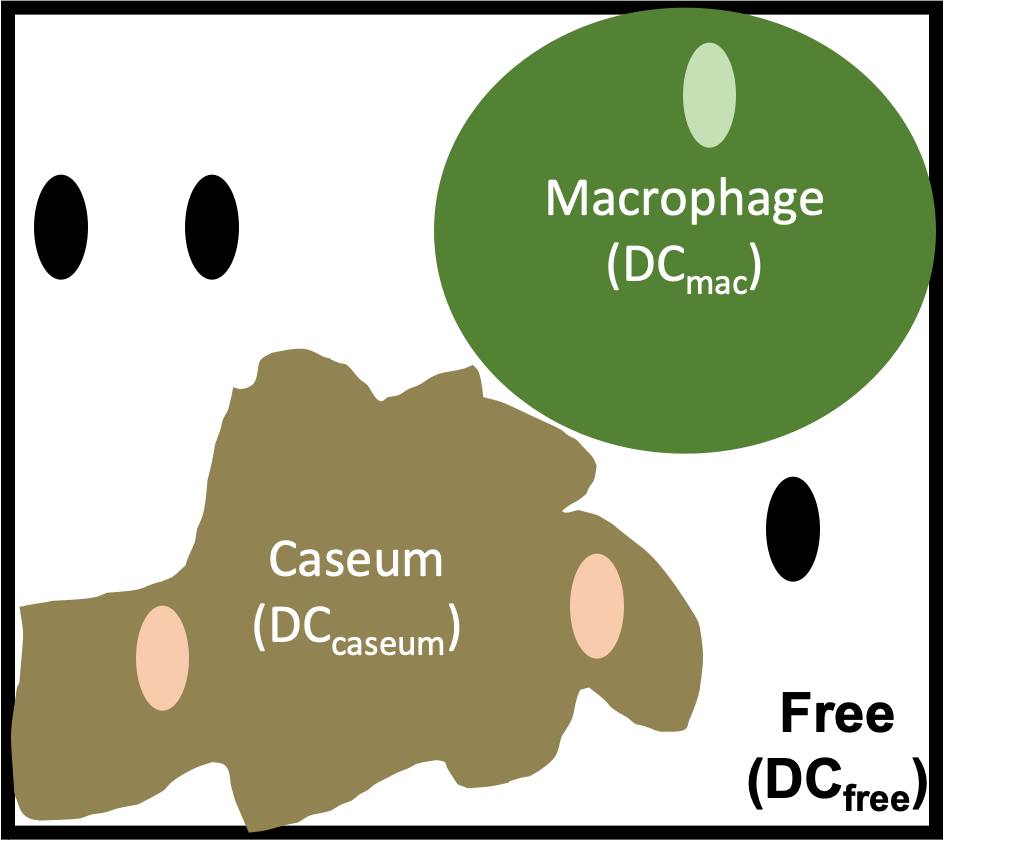


**Fig B.** **Schematic representation of how antibiotics are partitioned within a grid microcompartment within *GranSim*.** Within a microcompartment, antibiotics can be caseum-bound (*DC_caseum_*), be located within macrophages (*DC_mac_*) or be free without binding to anything (*DC_free_*), depending on the availability of caseous tissue and macrophages within that microgrid. (*DC_caseum_*: drug concentration bound to caseum, *DC_mac_*: drug concentration within a macrophage, *DC_free_*: free drug concentration, green circle: macrophage, brown bleb: caseum, black circles: replicating extracellular Mtb, light green circle: intracellular Mtb, tan circle: nonreplicating Mtb).

In the previous version of the PD model used in *GranSim*, the effective concentration that was used to calculate the killing rate constant (Eq.5) depended on the location of the bacteria. Namely, *DC_mac_* (drug concentration in a macrophage) and *DC_free_* (free concentration) were the effective concentrations to calculate the killing rate constants for intra- (light green circles in Fig B) and extracellular (replicating (black circles in Fig B) and non-replicating (tan circles in Fig B)) Mtb, respectively. However, the bactericidal assays we use to calibrate PD parameters (*E_max_*, *C_50_* and *h*) yields the fraction of Mtb that are killed based on the concentration of antibiotics Mtb were incubated with, i.e., the total concentration on the plate, rather than the partitioned concentration (*DC_mac_* or *DC_free_*). Therefore, we now use total concentration in the PD model such that the effective concentration is the total concentration on the microcompartment (*DC_total_*), namely:

${DC}_{total}=\frac{{DC}_{mac}{VOL}_{mac}+\left( {DC}_{free}+{DC}_{caseum} \right){VOL}_{grid}}{{VOL}_{grid}}$ (Eq.1)

where *VOL_mac_* and *VOL_grid_* are the volumes of a macrophage (4.85x10^-12^ liters) and the microgrid compartment in *GranSim* (8x10^-12^ liters), respectively.

1. Due to the changes in Point 2, we recalibrated the PK/PD for all drugs. We calibrated plasma and tissue PK parameters of H,R,Z and M to spatial and temporal human data [5] and E to rabbit data [6] (only moxifloxacin is shown in Fig C). We also calibrated the PD parameters of HRZEM to *in vitro* bactericidal assays (Table A).


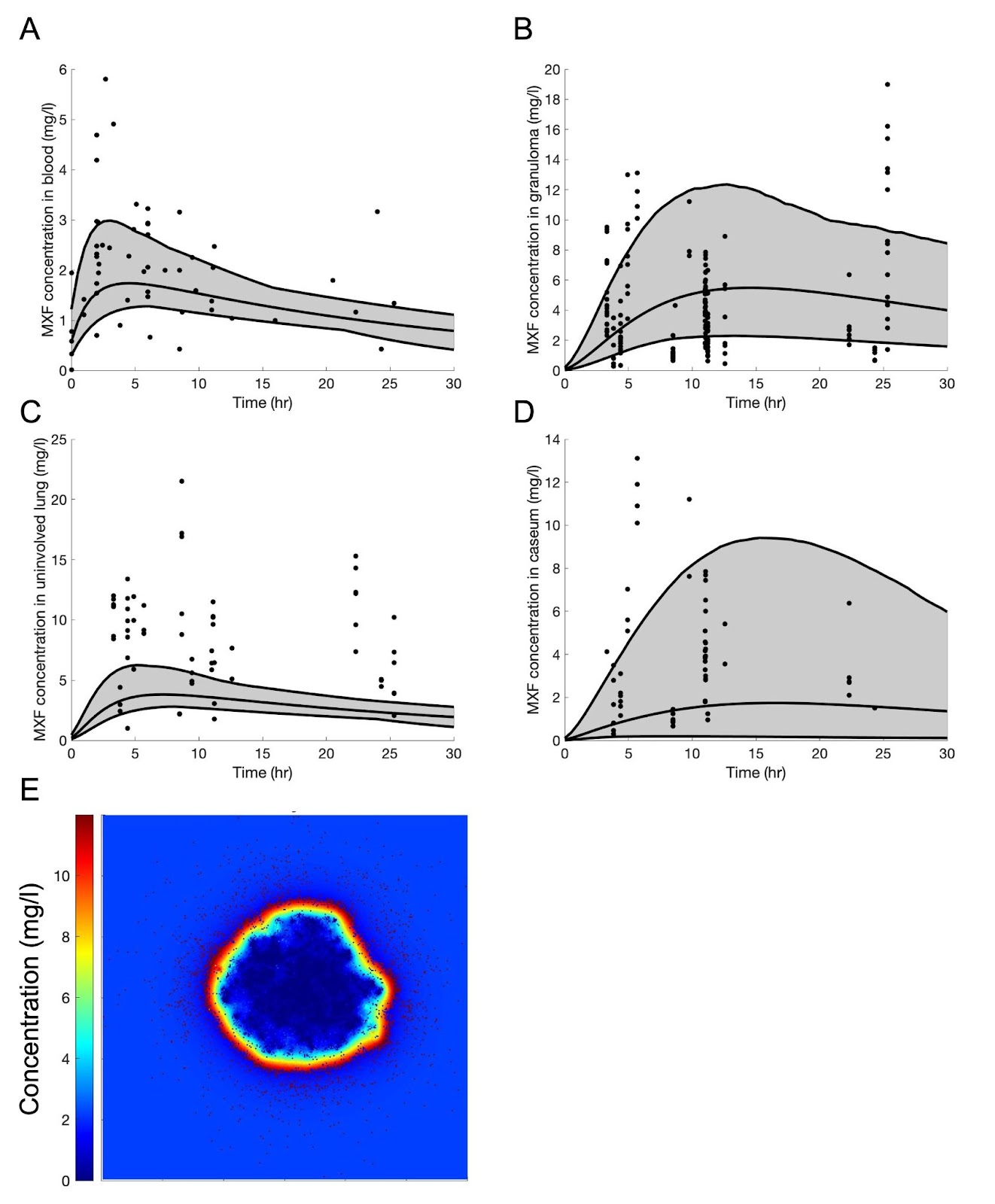


**Fig C.** **Calibration of *GranSim* PK/PD to Moxifloxacin datasets.** Calibration of moxifloxacin (MXF) plasma and tissue PK to temporal (black dots in A-D) and spatial (see Fig 2B in [5]) data from human granulomas. Black lines in A-D are the maximum, mean and minimum (from top to bottom) values of MXF concentrations resulting from 100 *GranSim* simulations, and the black shaded area is between the minimum and maximum. Average simulated MXF concentrations in (A) blood, (B) granuloma, (C) uninvolved lung and (D) caseum agree with human data. (E) Spatial analysis of how MXF is distributed within a granuloma in *GranSim* indicates that MXF does not easily diffuse into caseum, which is consistent with MALDI-MS imaging of granulomas in [5].  All other drugs were calibrated using this same approach.

**Table A.** **Pharmacodynamic (PD) parameters for each drug and Mtb type and sources for bactericidal assays used for calibration.** Emax values are reported as per *GranSim* timestep of 10 minutes.

| Drug name | Mtb type | E_max_ (1/timestep) | C_50_ (mg/l) | h | Reference |
| --- | --- | --- | --- | --- | --- |
| INH | Extracellular | 0.0082 | 0.012 | 1.8 | [7] |
|  | Intracellular | 0.0046 | 0.092 | 1.05 | [7] |
|  | Nonreplicating | 0.0021 | 1.86 | 1.29 | [8] |
| RIF | Extracellular | 0.045 | 0.081 | 2.14 | [9] |
|  | Intracellular | 0.012 | 0.6 | 0.4 | [9] |
|  | Nonreplicating | 0.0048 | 4.14 | 1.55 | [8] |
| EMB | Extracellular | 0.0091 | 0.5 | 3.45 | [10] |
|  | Intracellular | 0.0086 | 0.5 | 2.73 | [10] |
|  | Nonreplicating | 0 | 0 | 0 | [11] |
| PZA | Extracellular | 0.0019 | 13.66 | 0.94 | [12] |
|  | Intracellular | 0 | 0 | 0 | [12] |
|  | Nonreplicating | 0.43 | 11246.73 | 1.04 | [8] |
| MXF | Extracellular | 0.0074 | 0.06 | 4.99 | [13] |
|  | Intracellular | 0.0037 | 6.9 | 5.39 | [13] |
|  | Nonreplicating | 0.34 | 0.83 | 4.52 | [8] |

**References**

1. Cicchese JM, Sambarey A, Kirschner D, Linderman JJ, Chandrasekaran S. A multi-scale pipeline linking drug transcriptomics with pharmacokinetics predicts in vivo interactions of tuberculosis drugs. Sci Rep. 2021;11(1):5643. Epub 2021/03/11. doi: 10.1038/s41598-021-84827-0. PubMed PMID: 33707554; PubMed Central PMCID: PMCPMC7971003.

2. Joslyn LR, Linderman JJ, Kirschner DE. A virtual host model of Mycobacterium tuberculosis infection identifies early immune events as predictive of infection outcomes. J Theor Biol. 2022;539:111042. Epub 20220131. doi: 10.1016/j.jtbi.2022.111042. PubMed PMID: 35114195; PubMed Central PMCID: PMCPMC9169921.

3. Hult C, Mattila JT, Gideon HP, Linderman JJ, Kirschner DE. Neutrophil Dynamics Affect. Front Immunol. 2021;12:712457. Epub 20211005. doi: 10.3389/fimmu.2021.712457. PubMed PMID: 34675916; PubMed Central PMCID: PMCPMC8525425.

4. Sarathy JP, Liang HH, Weiner D, Gonzales J, Via LE, Dartois V. An In Vitro Caseum Binding Assay that Predicts Drug Penetration in Tuberculosis Lesions. J Vis Exp. 2017;(123). Epub 20170508. doi: 10.3791/55559. PubMed PMID: 28518128; PubMed Central PMCID: PMCPMC5607931.

5. Prideaux B, Via LE, Zimmerman MD, Eum S, Sarathy J, O'Brien P, et al. The association between sterilizing activity and drug distribution into tuberculosis lesions. Nat Med. 2015;21(10):1223-7. Epub 20150907. doi: 10.1038/nm.3937. PubMed PMID: 26343800; PubMed Central PMCID: PMCPMC4598290.

6. Zimmerman M, Lestner J, Prideaux B, O'Brien P, Dias-Freedman I, Chen C, et al. Ethambutol Partitioning in Tuberculous Pulmonary Lesions Explains Its Clinical Efficacy. Antimicrob Agents Chemother. 2017;61(9). Epub 20170824. doi: 10.1128/AAC.00924-17. PubMed PMID: 28696241; PubMed Central PMCID: PMCPMC5571334.

7. Jayaram R, Shandil RK, Gaonkar S, Kaur P, Suresh BL, Mahesh BN, et al. Isoniazid pharmacokinetics-pharmacodynamics in an aerosol infection model of tuberculosis. Antimicrob Agents Chemother. 2004;48(8):2951-7. doi: 10.1128/AAC.48.8.2951-2957.2004. PubMed PMID: 15273105; PubMed Central PMCID: PMCPMC478500.

8. Sarathy JP, Via LE, Weiner D, Blanc L, Boshoff H, Eugenin EA, et al. Extreme Drug Tolerance of Mycobacterium tuberculosis in Caseum. Antimicrob Agents Chemother. 2018;62(2). Epub 20180125. doi: 10.1128/AAC.02266-17. PubMed PMID: 29203492; PubMed Central PMCID: PMCPMC5786764.

9. Jayaram R, Gaonkar S, Kaur P, Suresh BL, Mahesh BN, Jayashree R, et al. Pharmacokinetics-pharmacodynamics of rifampin in an aerosol infection model of tuberculosis. Antimicrob Agents Chemother. 2003;47(7):2118-24. doi: 10.1128/AAC.47.7.2118-2124.2003. PubMed PMID: 12821456; PubMed Central PMCID: PMCPMC161844.

10. Hartkoorn RC, Chandler B, Owen A, Ward SA, Bertel Squire S, Back DJ, et al. Differential drug susceptibility of intracellular and extracellular tuberculosis, and the impact of P-glycoprotein. Tuberculosis (Edinb). 2007;87(3):248-55. Epub 20070126. doi: 10.1016/j.tube.2006.12.001. PubMed PMID: 17258938.

11. Lakshminarayana SB, Huat TB, Ho PC, Manjunatha UH, Dartois V, Dick T, et al. Comprehensive physicochemical, pharmacokinetic and activity profiling of anti-TB agents. J Antimicrob Chemother. 2015;70(3):857-67. Epub 20141111. doi: 10.1093/jac/dku457. PubMed PMID: 25587994; PubMed Central PMCID: PMCPMC7714050.

12. Zhang Y, Mitchison D. The curious characteristics of pyrazinamide: a review. Int J Tuberc Lung Dis. 2003;7(1):6-21. PubMed PMID: 12701830.

13. Shandil RK, Jayaram R, Kaur P, Gaonkar S, Suresh BL, Mahesh BN, et al. Moxifloxacin, ofloxacin, sparfloxacin, and ciprofloxacin against Mycobacterium tuberculosis: evaluation of in vitro and pharmacodynamic indices that best predict in vivo efficacy. Antimicrob Agents Chemother. 2007;51(2):576-82. Epub 20061204. doi: 10.1128/AAC.00414-06. PubMed PMID: 17145798; PubMed Central PMCID: PMCPMC1797767.
