# Supplementary material for: Optimizing tuberculosis treatment efficacy: Comparing the standard regimen with Moxifloxacin-containing regimens
Source: PLoS Comput Biol. 2023 Jun 15;19(6):e1010823. doi: 10.1371/journal.pcbi.1010823 (PMC10306236; doi:10.1371/journal.pcbi.1010823)
Supplement: Attachment — Submitted filename: response_to_reviewers_final_050323.docx [file pcbi.1010823.s003.docx]

*We thank the reviewers for the insightful comments that helped us greatly improve our manuscript. One general theme was the need for more clarity about the model structure itself. To address this, we created a new figure (Fig 8) representing the model dynamics that would hopefully address this.*

**Reviewer #1:** This is a very well written manuscript that integrates computational and experimental approaches to advance the field of TB therapeutics, by addressing some of the hardest problems in the field, reducing treatment duration and optimizing dose. I'm attaching some questions below for the authors to provide written response back on and help improve the manuscript from the standpoint of the reader.

Line 169/170: Can the model be extended to predict relapse rate in patients after end of treatment? Simulation results indicate one would expect a better outcome, but the translational results in the clinic indicate otherwise.

*We thank the reviewer for their comment. We also believe that predicting relapse rates is essential for predicting TB regimen efficacies. As we discussed in the last paragraph of the discussion (L 527-539), our granuloma-scale model, GranSim, limits our ability to simulate lymph nodes that are shown to induce reinfection or reactivation of the disease (Ganchua et al., Epub, 2020). We can conclude that infection will reactivate if there are any unsterilized granulomas left at the end of the treatment (see treatment with ZM and EM in S4C Fig). However, the opposite may not always be true. The disease can still relapse even if all granulomas sterilize by the end of the treatment due to lack of information on lymph node status. We are currently working on simulating treatments at a whole host-scale using our model HostSim (Joslyn et al., Epub, 2022) that includes lymph node dynamics and will enable us to predict relapse rates; however, this question is beyond the scope of our study. We acknowledge this issue is a limitation of our study.*

*For clarity, we added the following sentences in the manuscript (L 224-228):* ***“In the REMoxTB study, noninferiority of moxifloxacin-containing regimens was not shown due to the higher relapse rates. Our granuloma-scale model limits our ability to predict disease relapse because it does not contain the dynamics of Mtb-containing granulomas in lymph nodes, which have been shown to induce reinfection (see Discussion).”***

Line 243: What do you make of the differences between model and experimental results on metabolic effects of RMZE?

*The model results show that treatment with all regimens (HRZE, HMZE and RMZE) results in a decrease in the inflammatory activity compared to the control case. Different from the model results, experimental results show that only HMZE treatment decreases inflammation, whereas inflammation remains unchanged with HRZE and RMZE treatments. We made the following changes in that paragraph (L 265-269)****: “Similar to the FDG PET-CT results from NHP experiments, GranSim simulations demonstrated that treatment with HMZE decreases metabolic activity significantly (Fig 4B). However, GranSim predicted reduced inflammation with RMZE and HRZE as well, unlike NHP experiments, where no change was observed compared to the control.”***

Line 243: Are there markers of inflammation in the model and have these shown any results showing reduction in inflammation?

*Yes, there are markers of inflammation in GranSim, such as the cytokine TNF, infected macrophages, chronically infected macrophages (macrophages that have a high number of intracellular Mtb), activated macrophages, IFNγ-secreting T cells, and cytotoxic T cells. We derived the FDG avidity measurement in Figure 4B as a function of these markers of inflammation (see Methods- NHP granuloma FDG avidity measurement in GranSim). Therefore, decreased FDG avidity in GranSim results from the reduction of inflammation markers in GranSim. We clarified it in the text (L 262-265) as:* ***“In GranSim, we monitor metabolic activity of a granuloma based on cells associated with inflammation within granulomas, such as the number of various cell types and inflammatory measures of activity within granulomas (see Methods for more details).”***

Line 260: What other features of granulomas were varied in these simulations? e.g. size, inflammatory, metabolic,…

*To generate an in silico library of granulomas, we varied parameters that determine host immune responses during Mtb infection (e.g., immune cell recruitment, macrophage apoptosis rate etc.) and metabolic activity (e.g., chemokine/cytokine production rate) (see Table 1 in Cicchese et al., Front Pharm, 2020 for the full list of parameters that are varied). As a result of simulating GranSim with these parameter sets, granulomas emerge with varying dynamics of CFU/immune cell counts and granuloma sizes. We clarified this in the text (L 136-143) as:* ***“We varied parameters that determine a variety of host immune responses during Mtb infection (e.g., immune cell recruitment, macrophage apoptosis rate etc.) and metabolic activity (e.g., chemokine/cytokine production rate, carrying capacity of macrophages etc.) (see Table 1 in [8] for the full list of parameters that are varied). Then, we simulated three replications with each parameter set to capture both types of uncertainty present [48]. As a result of simulating GranSim with these parameter sets, granulomas emerge with variable dynamics of CFU/immune cell counts and granuloma sizes.”***

Line 351: What PKPD information about each drug was used to do the dose predictions, and have you considered penetration depth?

*Our PK model consists of a compartmental ODE model of plasma PK, penetration into the lung tissue (GranSim environment) through vascular sources, diffusion through tissue, binding to caseum /epithelium and partitioning into macrophages (see L 611-617). We added an explanatory figure in the manuscript (Fig 8).*

*Penetration depth is controlled by processes like diffusion, binding to caseum/epithelial tissue and drug uptake by macrophages. We clarified this in the text (L 615-618) as:* ***“We then calculated diffusion through tissue, binding to caseum and epithelium and partitioning into macrophages [6, 8, 38, 75]. These processes control penetration depth, i.e., how deeply antibiotics penetrate into granulomas.”***

*For each antibiotic, we calibrate parameters that govern these PK processes to drug concentrations in plasma, uninvolved lung, lesion and caseum from humans, NHPs or rabbits. We elaborated this issue in the main text (L 671-674) as: “****To calibrate the PK parameters of each antibiotic in GranSim, we used antibiotic concentrations at various time points and tissue types (e.g., plasma, uninvolved lung, caseum and lesion) from human or animal samples after administering human-equivalent doses (see S9 Fig for moxifloxacin PK calibration).****”*

*In GranSim, we have 3 types of bacteria: intracellular, replicating extracellular and nonreplicating extracellular Mtb. We model PD by using a Hill function that determines the concentration dependent antibiotic killing rate constant (Fig 8). The parameters of the Hill function are calibrated separately to each bacterial phenotype (by location) and each antibiotic using in vitro or ex vivo bactericidal assays. We use bactericidal assays of infected macrophages, Mtb in rich growth media and Mtb in caseum mimic for the PD of intracellular, replicating-extracellular and nonreplicating Mtb, respectively (see L 619-631).*

Discussion: Future work can consider testing a subset of the top functioning combinations in an animal model. Also consider expanding the model to include prediction of clinical relapse.

*We thank the reviewer for their key insights. These are indeed great points that we consider. We mentioned that we aim to inform experiments with our predictions in L 536-538. We elaborated this idea and added* ***“In current and future work, we aim to test the most efficacious regimens in an animal model to validate our predictions.”*** *in L 538-539.*

*We already mentioned above that “we are currently adapting our host-scale TB model, HostSim” (L 530) to include the prediction of clinical relapse in L 532-536.*

**Reviewer #2:** 1. Please clarify how did the authors derive adjusted concentration for 3 or 4 drug combinations?

*We thank the reviewer for their comment. For simplicity, we derived the 2-drug combination case in the manuscript, but we understand the confusion. We generalized that section in the methods (L 649-668) for a case with n drug combinations.*

2. With respect to the simulations with GranSim shown in Figure 6: As these were standard doses, and some of the combinations have been evaluated in literature in preclinical models, is there a similarity in trends for time to sterilization metric? Something like that could lend validity to the GranSim simulations.

*We thank the reviewer for their insightful comment, this is indeed a great idea. We added the sentence To L 348-350:* ***“Our results agree with preclinical studies on mouse models in that HRZM [40, 55], RMZE [55] and RMZ [39, 40] sterilize mice faster than HRZE.”*** *We would like to point out that we can only compare the relative performances of regimens rather than the absolute values of regimens’ sterilization times, as these preclinical mouse studies report the sterilization of the whole lung, unlike GranSim’s granuloma-scale measures.*

3. Comparing GranSim simulations to NHP and humans (Figure 2,3): Does GranSim consider interspecies PK or PD parameter scaling, or differences in PD parameter within granulomas of the different species?

*We calibrate the PK parameters of each antibiotic to concentrations at various time points and tissue types (e.g., plasma, uninvolved lung, caseum and lesion) from human or rabbit/NHP animal samples after administering human-equivalent doses. Therefore, PK parameters don’t need to be scaled. We clarified this issue in L 671-674 as* ***“To calibrate the PK parameters of each antibiotic in GranSim, we used antibiotic concentrations at various time points and tissue types (e.g., plasma, uninvolved lung, caseum and lesion) from human or rabbit/NHP animal samples after administering human-equivalent doses (see S9 Fig for moxifloxacin PK calibration).”***

*We do not scale PD parameters for different species. However, GranSim has 3 distinct Mtb subpopulations that are found in NHP granulomas (intracellular in macrophages, replicating-extracellular and nonreplicating trapped in caseum) and we calibrate PD parameters of each Mtb subpopulation using bactericidal assays that are performed in microenvironments relevant to NHP granulomas. We modified the manuscript to clarify this issue in L 586-588 as* ***"To mimic that of actual infection in NHPs, we simulate three distinct subpopulations of Mtb based on their location within granulomas..."*** *and L 627-631* ***"The calibration is based on bactericidal assays of Mtb within microenvironments that are relevant to NHP granulomas, namely infected macrophages [18, 19, 78-80], Mtb in rich growth media [18, 19, 78-80] and Mtb in caseum mimic [20], for intracellular, replicating-extracellular and Mtb in caseum, respectively (Fig 8D).".***

4. In Figure 1, Does each individual data point come from single NHP or multiple NHPs? Please elaborate?

*Each individual data point comes from a granuloma of a single NHP. Multiple data points at the same time step may come from the same NHP though, if NHPs have multiple granulomas by the time they were necropsied. We elaborated this issue at L 167-172 as* ***“Each curve represents a single granuloma simulation with a single parameter set using GranSim, and black dots are CFU counts from NHP granulomas [46, 47]. Each individual data point comes from a granuloma of a unique NHP; however, multiple data points at the same time step may come from the same NHP if that NHP has developed multiple granulomas by the time they were necropsied.*** ***In total, the data points come from 42 monkeys and 646 granulomas, and each monkey has 2-40 granulomas (the median is 14.5, 25th and 75th percentiles are 9 and 20, respectively.).”***

5. With respect to Figure 5, what is the typical distribution of the ratio of high-low CFU granulomas observed clinically? Clinical relevance could add value.

*To our knowledge, there are no granuloma-scale data on CFU counts from human samples. The animal model that closest resembles human TB is the NHP model, and we have CFU counts from NHP granulomas that we showed in Fig 1. These data suggest that 70% of the unsterilized granulomas are low-CFU, whereas 30% of these are high-CFU. However, high-CFU granulomas are harder to treat and likely result in an active disease. Therefore, we believe that investigating high-CFU granulomas is valuable. We elaborated this issue in L 288-294* ***“We used equal sample sizes (N=100) for high- and low-CFU granulomas for a fairer comparison. However, data from NHP granulomas (black dots in Fig 1) suggest that 30% of unsterilized granulomas are high-CFU, while 70% of them are low-CFU granulomas. Although low-CFU granulomas are clinically more common as most individuals have latent infection, high-CFU granulomas are harder to treat and likely result in an active disease. Therefore, it is crucial to understand drug dynamics within high-CFU granulomas.”.***

6. When making the various comparisons, particularly in figures 2-6, provide a measure of how much change was observed along with a measure of variability and a statistic quantifying the differences.

*We assessed each regimen’s efficacy with a granuloma-scale measure “sterilization time” that quantifies the length of the treatment needed to clear all Mtb within a granuloma (Fig 2C, 3A, 5D-F, S3D-F Fig and S4D-F Fig). We also assessed the metabolic activity of granulomas with FDG avidity measure in Fig 4B.*

*By visualizing each measure with a box plot, we provided the median (central line of the box), 25^th^ and 75^th^ (the bottom and top edges of the box, respectively) percentiles as a measure of variability. We clarified this issue in the captions of Figs 2-6, S3 Fig and S4 Fig as* ***“The central red lines in box plots represent the median, whereas the bottom and the top edges of boxes represent 25th and 75th percentiles, respectively.”****.*

*To assess statistical significance of the differences, we used one-tailed paired t-test in Fig 3A and 4B. We added the same test to Fig 2C, Fig 5, S3 Fig and S4 Fig. We would like to note that we only tested the statistical significance between regimens with and without moxifloxacin for simplicity in Fig 5, S3 Fig and S4 Fig. We clarified this in the caption of Fig 5 as* ***“Significance test was performed only between HRZE and moxifloxacin-containing regimens (*p<0.0001, one-tailed paired t-test).”*** *and in the appendix in the captions of S3 Fig and S4 Fig as* ***“We performed significance tests between each possible pair of regimens with (red boxes) and without (black boxes) including moxifloxacin ...”***

*We modified Fig 6 and determined the regimens that have significantly lower sterilization times than HRZE with one-tailed paired t-test, unlike before where we only compared the average sterilization times without statistical analysis. We plotted these regimens with significantly lower sterilization times than HRZE in color in panels A-C. We also added the sterilization times of these regimens in panels D-F. We modified the caption of Fig 6 as* ***“Comparison of (A-C) sterilizing rates and (D-F) sterilization times of all regimen combinations of HRZEM to HRZE (thick green curve in all panels) in (A and D) 100 high-CFU, (B and E) 100 low-CFU and (C and F) a combination of 100 high- and 100 low-CFU granulomas.”.***
